# 4-Chloro-*N*-cyclo­hexyl­benzene­sulfonamide

**DOI:** 10.1107/S1600536812001870

**Published:** 2012-01-21

**Authors:** Shahzad Sharif, Shumaila Younas Mughal, Islam Ullah Khan, Mehmet Akkurt, Muneeb Hayat Khan

**Affiliations:** a Materials Chemistry Laboratory, Department of Chemistry, Government College University, Lahore 54000, Pakistan; b Department of Physics, Faculty of Sciences, Erciyes University, 38039 Kayseri, Turkey; c Materials Chemistry Laboratory, Department of Chemistry, Government College University, Lahore 54000, Pakistan, and, Pakistan and Punjab Forensic Science Agency, Thokar Niaz Baig, Lahore, Pakistan

## Abstract

The title compound, C_12_H_16_ClNO_2_S, adopts an L-shaped conformation, with the central C—S—N—C torsion angle being −78.0 (2)°. The cyclo­hexyl ring adopts a chair conformation. In the crystal, adjacent mol­ecules are connected by pairs of N—H⋯O hydrogen bonds around an inversion centre, forming cyclic dimers [graph set *R*_2_^2^(8)].

## Related literature

For background to the pharmacological uses of sulfonamides, see: Korolkovas (1988 ▶); Mandell & Sande (1992 ▶). For related structures, see: Sharif *et al.* (2011 ▶); Khan *et al.* (2010 ▶); John *et al.* (2010 ▶). For ring conformational analysis, see: Cremer & Pople (1975 ▶).
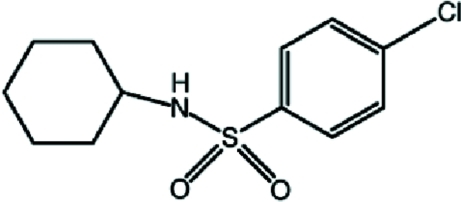


## Experimental

### 

#### Crystal data

C_12_H_16_ClNO_2_S
*M* *_r_* = 273.78
Monoclinic, 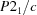

*a* = 11.1226 (5) Å
*b* = 6.2490 (2) Å
*c* = 19.8635 (9) Å
β = 96.505 (2)°
*V* = 1371.73 (10) Å^3^
*Z* = 4
Mo *K*α radiation
μ = 0.42 mm^−1^
*T* = 296 K
0.29 × 0.15 × 0.11 mm

#### Data collection

Bruker APEXII CCD diffractometer
12714 measured reflections
3365 independent reflections
2075 reflections with *I* > 2σ(*I*)
*R*_int_ = 0.030

#### Refinement

*R*[*F* ^2^ > 2σ(*F* ^2^)] = 0.053
*wR*(*F* ^2^) = 0.150
*S* = 1.03
3365 reflections
157 parameters
1 restraint
H atoms treated by a mixture of independent and constrained refinement
Δρ_max_ = 0.39 e Å^−3^
Δρ_min_ = −0.25 e Å^−3^

### 

Data collection: *APEX2* (Bruker, 2007 ▶); cell refinement: *SAINT* (Bruker, 2007 ▶); data reduction: *SAINT*; program(s) used to solve structure: *SIR97* (Altomare *et al.*, 1999 ▶); program(s) used to refine structure: *SHELXL97* (Sheldrick, 2008 ▶); molecular graphics: *ORTEP-3* for Windows (Farrugia, 1997 ▶); software used to prepare material for publication: *WinGX* (Farrugia, 1999 ▶) and *PLATON* (Spek, 2009 ▶).

## Supplementary Material

Crystal structure: contains datablock(s) global, I. DOI: 10.1107/S1600536812001870/hg5164sup1.cif

Structure factors: contains datablock(s) I. DOI: 10.1107/S1600536812001870/hg5164Isup2.hkl

Supplementary material file. DOI: 10.1107/S1600536812001870/hg5164Isup3.cml

Additional supplementary materials:  crystallographic information; 3D view; checkCIF report

## Figures and Tables

**Table 1:** Hydrogen-bond geometry (Å, °)

*D*—H⋯*A*	*D*—H	H⋯*A*	*D*⋯*A*	*D*—H⋯*A*
N1—H1*N*⋯O1^i^	0.85 (4)	2.05 (4)	2.891 (4)	170 (3)

Symmetry code: (i) 
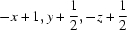
.

**Fig. 1.**
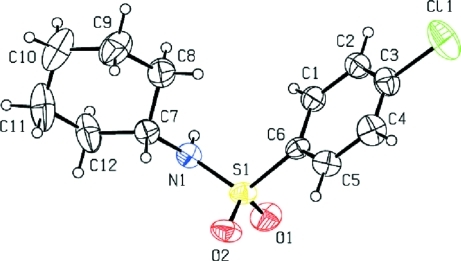
The title molecule, showing the atom numbering scheme. Displacement ellipsoids for non-H atoms are drawn at the 30% probability level.

**Fig. 2.**
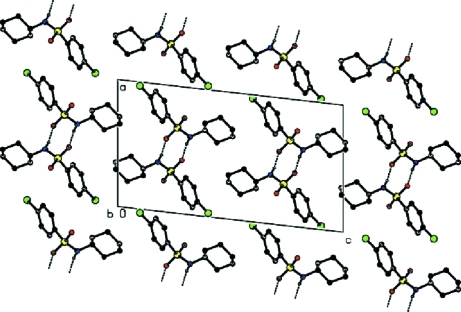
The crystal packing of (I) down b axis, showing the molecules are linked into dimers by pairs of N—H ··· O hydrogen bonds, forming R22(8) graph-set motif. Hydrogen atoms not involved in hydrogen bonding have been omitted for clarity.

## supplementary crystallographic information

### Comment

Sulfonamide group containing drugs are extensively used for the treatment of certain infections caused by Gram-positive and Gram-negative microorganisms (Korolkovas, 1988; Mandell & Sande, 1992). In continuation of our on going structural studies of cyclohexylamine and sulfonamides synthesis (John *et al.*, 2010; Khan *et al.*, 2010; Sharif *et al.*, 2011), herein the crystal structure of title compound (I) is described.

In (I), (Fig. 1), the S atom has a distorted tetrahedral geometry within a CNO_2_ donor set [maximum deviation: O—S—O = 119.45 (12)°]. The central C6–S1–N1–C7 torsion angle is -78.0 (2)°. The C7–C12 cyclohexyl ring adopts a chair conformation, with puckering parameters (Cremer & Pople, 1975) Q = 0.536 (4) Å, θ = 180.0 (4) °, φ = 196 (16) °.

In the crystal, two adjacent molecules are linked by a pair of N—H···O hydrogen bonds, forming an inversion dimer with an *R*_2_^2^(8) ring motif (Table 1, Fig. 2).

### Experimental

To 115 µl (1 mmol) of cyclohexylamine in 10 ml distilled water, was added 211 mg (1 mmol) of 4-chlorobenzenesulfonyl chloride while maintaining the pH of reaction mixture at 8 by using 3% sodium carbonate solution. Consumption of the reactants was confirmed by TLC. The pH of reaction mixture was adjusted by 3 N HCl at 3. Precipitates formed, washed with water and crystallized from methanol.

### Refinement

The NH H–atom was located in a difference Fourier map and isotropically refined with a distance restraint: N—H = 0.86 (2) Å. C-bound H atoms were placed in calculated positions with C—H distances in the range 0.93–0.98 Å and and were refined using a riding model with *U*_iso_(H) = 1.2*U*_eq_(C). In the final refinement two low angle reflections evidently effected by the beam stop were omitted, *i.e.* 0 0 2 and 1 0 0.

### Crystal data

**Table:**

C_12_H_16_ClNO_2_S	*F*(000) = 576
*M**_r_* = 273.78	*D*_x_ = 1.326 Mg m^−^^3^
Monoclinic, *P*2_1_/*c*	Mo *K*α radiation, λ = 0.71069 Å
Hall symbol: -P 2ybc	Cell parameters from 3062 reflections
*a* = 11.1226 (5) Å	θ = 2.6–21.6°
*b* = 6.2490 (2) Å	µ = 0.42 mm^−^^1^
*c* = 19.8635 (9) Å	*T* = 296 K
β = 96.505 (2)°	Needle, light brown
*V* = 1371.73 (10) Å^3^	0.29 × 0.15 × 0.11 mm
*Z* = 4	

### Data collection

**Table:**

Bruker APEXII CCD diffractometer	2075 reflections with *I* > 2σ(*I*)
Radiation source: sealed tube	*R*_int_ = 0.030
graphite	θ_max_ = 28.3°, θ_min_ = 3.4°
φ and ω scans	*h* = −14→14
12714 measured reflections	*k* = −6→8
3365 independent reflections	*l* = −26→26

### Refinement

**Table:**

Refinement on *F*^2^	Primary atom site location: structure-invariant direct methods
Least-squares matrix: full	Secondary atom site location: difference Fourier map
*R*[*F*^2^ > 2σ(*F*^2^)] = 0.053	Hydrogen site location: inferred from neighbouring sites
*wR*(*F*^2^) = 0.150	H atoms treated by a mixture of independent and constrained refinement
*S* = 1.03	*w* = 1/[σ^2^(*F*_o_^2^) + (0.0613*P*)^2^ + 0.526*P*] where *P* = (*F*_o_^2^ + 2*F*_c_^2^)/3
3365 reflections	(Δ/σ)_max_ < 0.001
157 parameters	Δρ_max_ = 0.39 e Å^−^^3^
1 restraint	Δρ_min_ = −0.24 e Å^−^^3^

### Special details

**Table:**

Geometry. Bond distances, angles *etc*. have been calculated using the rounded fractional coordinates. All su's are estimated from the variances of the (full) variance-covariance matrix. The cell e.s.d.'s are taken into account in the estimation of distances, angles and torsion angles
Refinement. Refinement on *F*^2^ for ALL reflections except those flagged by the user for potential systematic errors. Weighted *R*-factors *wR* and all goodnesses of fit *S* are based on *F*^2^, conventional *R*-factors *R* are based on *F*, with *F* set to zero for negative *F*^2^. The observed criterion of *F*^2^ > σ(*F*^2^) is used only for calculating -*R*-factor-obs *etc*. and is not relevant to the choice of reflections for refinement. *R*-factors based on *F*^2^ are statistically about twice as large as those based on *F*, and *R*-factors based on ALL data will be even larger.

### Fractional atomic coordinates and isotropic or equivalent isotropic displacement parameters (Å^2^)

**Table:**

	*x*	*y*	*z*	*U*_iso_*/*U*_eq_	
Cl1	0.97047 (9)	0.63719 (19)	0.09885 (6)	0.1159 (5)	
S1	0.67445 (5)	0.02579 (10)	0.26455 (3)	0.0538 (2)	
O1	0.57744 (18)	−0.0412 (3)	0.21563 (11)	0.0788 (8)	
O2	0.75531 (17)	−0.1301 (3)	0.29647 (11)	0.0710 (7)	
N1	0.61305 (18)	0.1518 (3)	0.32146 (11)	0.0527 (7)	
C1	0.7127 (2)	0.3981 (4)	0.19816 (13)	0.0581 (9)	
C2	0.7777 (3)	0.5312 (4)	0.16141 (14)	0.0650 (9)	
C3	0.8901 (3)	0.4698 (5)	0.14686 (14)	0.0654 (10)	
C4	0.9396 (2)	0.2788 (5)	0.16961 (16)	0.0733 (11)	
C5	0.8750 (2)	0.1454 (4)	0.20712 (14)	0.0606 (9)	
C6	0.7611 (2)	0.2037 (4)	0.22098 (12)	0.0465 (7)	
C7	0.6789 (2)	0.2145 (4)	0.38711 (12)	0.0541 (8)	
C8	0.7329 (3)	0.4325 (5)	0.38666 (16)	0.0829 (11)	
C9	0.7924 (4)	0.4979 (7)	0.45609 (18)	0.1069 (17)	
C10	0.7084 (4)	0.4802 (9)	0.5084 (2)	0.117 (2)	
C11	0.6528 (5)	0.2674 (10)	0.50951 (18)	0.135 (2)	
C12	0.5920 (4)	0.1970 (7)	0.44002 (17)	0.1033 (16)	
H1	0.63600	0.43810	0.20780	0.0700*	
H1N	0.555 (3)	0.234 (6)	0.3060 (19)	0.1390*	
H2	0.74580	0.66260	0.14640	0.0780*	
H4	1.01630	0.23970	0.15970	0.0880*	
H5	0.90830	0.01600	0.22310	0.0730*	
H7	0.74450	0.11150	0.39850	0.0650*	
H8A	0.67010	0.53500	0.37150	0.0990*	
H8B	0.79270	0.43550	0.35470	0.0990*	
H9A	0.86230	0.40740	0.46850	0.1280*	
H9B	0.82060	0.64450	0.45420	0.1280*	
H10A	0.75200	0.51000	0.55240	0.1410*	
H10B	0.64520	0.58700	0.49970	0.1410*	
H11A	0.59300	0.26840	0.54140	0.1620*	
H11B	0.71460	0.16380	0.52540	0.1620*	
H12A	0.56470	0.05020	0.44260	0.1240*	
H12B	0.52180	0.28630	0.42710	0.1240*	

### Atomic displacement parameters (Å^2^)

**Table:**

	*U*^11^	*U*^22^	*U*^33^	*U*^12^	*U*^13^	*U*^23^
Cl1	0.0867 (7)	0.1367 (9)	0.1293 (9)	−0.0197 (6)	0.0339 (6)	0.0505 (7)
S1	0.0456 (4)	0.0438 (3)	0.0725 (4)	−0.0069 (3)	0.0087 (3)	−0.0065 (3)
O1	0.0634 (12)	0.0815 (13)	0.0904 (14)	−0.0310 (10)	0.0034 (11)	−0.0211 (11)
O2	0.0691 (12)	0.0436 (9)	0.1030 (15)	0.0083 (9)	0.0210 (11)	0.0082 (9)
N1	0.0393 (11)	0.0562 (12)	0.0629 (13)	0.0041 (9)	0.0071 (10)	0.0035 (10)
C1	0.0540 (15)	0.0589 (15)	0.0637 (16)	0.0058 (13)	0.0169 (13)	0.0006 (12)
C2	0.0704 (18)	0.0594 (15)	0.0664 (16)	0.0034 (14)	0.0132 (15)	0.0056 (13)
C3	0.0553 (16)	0.0765 (18)	0.0646 (16)	−0.0132 (15)	0.0076 (13)	0.0050 (14)
C4	0.0398 (14)	0.091 (2)	0.090 (2)	−0.0036 (15)	0.0118 (15)	0.0011 (18)
C5	0.0418 (14)	0.0602 (15)	0.0793 (18)	0.0021 (12)	0.0048 (13)	0.0025 (13)
C6	0.0410 (12)	0.0461 (12)	0.0514 (13)	−0.0033 (10)	0.0015 (10)	−0.0093 (10)
C7	0.0463 (14)	0.0565 (14)	0.0597 (15)	0.0112 (12)	0.0063 (12)	0.0059 (12)
C8	0.095 (2)	0.082 (2)	0.0727 (19)	−0.0204 (19)	0.0134 (18)	−0.0077 (16)
C9	0.106 (3)	0.119 (3)	0.095 (3)	−0.014 (2)	0.009 (2)	−0.038 (2)
C10	0.092 (3)	0.178 (5)	0.082 (2)	0.019 (3)	0.009 (2)	−0.049 (3)
C11	0.132 (4)	0.216 (6)	0.059 (2)	−0.014 (4)	0.018 (2)	0.022 (3)
C12	0.100 (3)	0.141 (3)	0.072 (2)	−0.027 (2)	0.023 (2)	0.021 (2)

### Geometric parameters (Å, °)

**Table:**

Cl1—C3	1.731 (4)	C10—C11	1.468 (8)
S1—O1	1.431 (2)	C11—C12	1.531 (6)
S1—O2	1.425 (2)	C1—H1	0.9300
S1—N1	1.594 (2)	C2—H2	0.9300
S1—C6	1.762 (3)	C4—H4	0.9300
N1—C7	1.475 (3)	C5—H5	0.9300
N1—H1N	0.85 (4)	C7—H7	0.9800
C1—C2	1.366 (4)	C8—H8A	0.9700
C1—C6	1.384 (4)	C8—H8B	0.9700
C2—C3	1.370 (5)	C9—H9A	0.9700
C3—C4	1.370 (4)	C9—H9B	0.9700
C4—C5	1.374 (4)	C10—H10A	0.9700
C5—C6	1.376 (3)	C10—H10B	0.9700
C7—C12	1.510 (5)	C11—H11A	0.9700
C7—C8	1.489 (4)	C11—H11B	0.9700
C8—C9	1.517 (5)	C12—H12A	0.9700
C9—C10	1.478 (6)	C12—H12B	0.9700
			
O1—S1—O2	119.45 (12)	C5—C4—H4	120.00
O1—S1—N1	106.00 (12)	C4—C5—H5	120.00
O1—S1—C6	105.28 (12)	C6—C5—H5	120.00
O2—S1—N1	108.77 (12)	N1—C7—H7	108.00
O2—S1—C6	107.29 (11)	C8—C7—H7	108.00
N1—S1—C6	109.82 (11)	C12—C7—H7	108.00
S1—N1—C7	123.29 (16)	C7—C8—H8A	109.00
S1—N1—H1N	114 (3)	C7—C8—H8B	109.00
C7—N1—H1N	116 (3)	C9—C8—H8A	109.00
C2—C1—C6	120.0 (2)	C9—C8—H8B	109.00
C1—C2—C3	119.6 (3)	H8A—C8—H8B	108.00
Cl1—C3—C2	119.2 (2)	C8—C9—H9A	109.00
C2—C3—C4	121.1 (3)	C8—C9—H9B	109.00
Cl1—C3—C4	119.7 (2)	C10—C9—H9A	109.00
C3—C4—C5	119.5 (2)	C10—C9—H9B	109.00
C4—C5—C6	119.9 (2)	H9A—C9—H9B	108.00
S1—C6—C1	120.05 (17)	C9—C10—H10A	109.00
S1—C6—C5	119.92 (19)	C9—C10—H10B	109.00
C1—C6—C5	120.0 (2)	C11—C10—H10A	109.00
N1—C7—C8	113.5 (2)	C11—C10—H10B	109.00
C8—C7—C12	111.2 (3)	H10A—C10—H10B	108.00
N1—C7—C12	107.7 (2)	C10—C11—H11A	109.00
C7—C8—C9	112.1 (3)	C10—C11—H11B	109.00
C8—C9—C10	111.9 (4)	C12—C11—H11A	109.00
C9—C10—C11	112.3 (4)	C12—C11—H11B	109.00
C10—C11—C12	113.0 (4)	H11A—C11—H11B	108.00
C7—C12—C11	110.8 (4)	C7—C12—H12A	110.00
C2—C1—H1	120.00	C7—C12—H12B	109.00
C6—C1—H1	120.00	C11—C12—H12A	109.00
C1—C2—H2	120.00	C11—C12—H12B	109.00
C3—C2—H2	120.00	H12A—C12—H12B	108.00
C3—C4—H4	120.00		
			
O1—S1—N1—C7	168.78 (18)	C1—C2—C3—Cl1	178.7 (2)
O2—S1—N1—C7	39.2 (2)	Cl1—C3—C4—C5	−179.3 (2)
C6—S1—N1—C7	−78.0 (2)	C2—C3—C4—C5	0.6 (5)
N1—S1—C6—C5	135.6 (2)	C3—C4—C5—C6	0.6 (4)
O2—S1—C6—C1	−165.3 (2)	C4—C5—C6—S1	176.1 (2)
N1—S1—C6—C1	−47.2 (2)	C4—C5—C6—C1	−1.1 (4)
O1—S1—C6—C1	66.5 (2)	N1—C7—C8—C9	176.1 (3)
O2—S1—C6—C5	17.5 (2)	C12—C7—C8—C9	54.6 (4)
O1—S1—C6—C5	−110.7 (2)	N1—C7—C12—C11	−178.0 (3)
S1—N1—C7—C12	−144.8 (2)	C8—C7—C12—C11	−53.2 (4)
S1—N1—C7—C8	91.8 (2)	C7—C8—C9—C10	−54.4 (4)
C6—C1—C2—C3	0.7 (4)	C8—C9—C10—C11	53.3 (5)
C2—C1—C6—S1	−176.7 (2)	C9—C10—C11—C12	−53.2 (6)
C2—C1—C6—C5	0.5 (4)	C10—C11—C12—C7	53.0 (5)
C1—C2—C3—C4	−1.3 (4)		

### Hydrogen-bond geometry (Å, °)

**Table:**

*D*—H···*A*	*D*—H	H···*A*	*D*···*A*	*D*—H···*A*
N1—H1N···O1^i^	0.85 (4)	2.05 (4)	2.891 (4)	170 (3)

Symmetry codes: (i) −*x*+1, *y*+1/2, −*z*+1/2.
